# RNA N6-methyladenosine methylation in influenza A virus infection

**DOI:** 10.3389/fmicb.2024.1401997

**Published:** 2024-06-18

**Authors:** Xueer Liu, Weiqiang Chen, Kangsheng Li, Jiangtao Sheng

**Affiliations:** ^1^Department of Microbiology and Immunology, Guangdong Provincial Key Laboratory of Infectious Disease and Molecular Immunopathology, Shantou University Medical College, Shantou, Guangdong, China; ^2^Department of Neurosurgery, First Affiliated Hospital of Shantou University Medical College, Shantou, Guangdong, China

**Keywords:** influenza A virus, RNA modification, m^6^A methylation, immune escape, anti-IAV strategy

## Abstract

Influenza A virus (IAV) is a negative-sense single-stranded RNA virus that causes acute lung injury and acute respiratory distress syndrome, posing a serious threat to both animal and human health. N6-methyladenosine (m^6^A), a prevalent and abundant post-transcriptional methylation of RNA in eukaryotes, plays a crucial regulatory role in IAV infection by altering viral RNA and cellular transcripts to affect viral infection and the host immune response. This review focuses on the molecular mechanisms underlying m^6^A modification and its regulatory function in the context of IAV infection and the host immune response. This will provide a better understanding of virus–host interactions and offer insights into potential anti-IAV strategies.

## 1 Introduction

Influenza A virus (IAV) is a pathogen that primarily affects the respiratory system, often leading to seasonal influenza outbreaks and global pandemics. In severe cases, the virus can cause acute respiratory distress syndrome (ARDS), septic shock, and multiple organ failure (MOF), which can be life-threatening (Taubenberger and Kash, 2010; Worobey et al., 2014; Herold et al., 2015). The World Health Organization (WHO) estimates that influenza is responsible for ~1 billion infections worldwide each year, resulting in 3–5 million severe cases and up to 650,000 deaths from respiratory illness (World Health Organization, 2023). IAV belongs to the Orthomyxoviridae family and is an enveloped virus with a single-stranded negative-sense RNA genome (Chou et al., 2012). Its genome is composed of eight fragments that encode three viral polymerase proteins (PB1, PB2, and PA), hemagglutinin (HA), nucleoprotein (NP), neuraminidase (NA), matrix protein (M), and non-structural (NS) protein (McGeoch et al., 1976; Cho et al., 2020). The HA protein recognizes sialic acid receptors on the host cell surface, facilitating virus attachment and entry through endocytosis (Skehel and Wiley, 2000; Fan et al., 2019). Within the cytoplasm, viral proteins and RNAs are synthesized by viral polymerase catalysis and assembled into mature viral particles using various host factors that enhance polymerase activity (Te Velthuis and Fodor, 2016; Peacock et al., 2019; Carrique et al., 2020). Subsequently, the NA protein cleaves the glycosidic bonds between viral particles and the host cell membrane, initiating virion budding and completing the replication cycle of IAV (Mora et al., 2002; Yang et al., 2016). During the process of viral replication, both double-stranded and single-stranded RNAs are generated. These RNAs act as pathogen-associated pattern molecules (PAMPs) that recognize and bind to pattern recognition receptors (PRRs) in the host, activating innate immune responses (Brennan and Bowie, 2010; Yoo et al., 2013; Downey et al., 2017). This leads to the production of various cytokines and antiviral molecules that inhibit viral replication and help control infection.

The pathogenesis of IAV involves two main processes: direct damage caused by viral replication within host cells and indirect damage caused by a virus-induced cytokine storm (Liu et al., 2016; Zhang H. et al., 2022). Existing strains of the virus have developed resistance to anti-IAV drugs, and current IAV vaccines often do not provide sufficient protection against new and emerging strains during local and global pandemics (Gubareva et al., 2000; Ann et al., 2012). Therefore, there is an urgent need to identify new targets and develop effective drugs for the control of influenza infections.

The discovery of various chemical modifications to RNA, such as the methylation and acetylation of bases such as adenine, guanine, and cytosine, has increased in recent years. Among these modifications, methylation is considered to be the most significant (Saletore et al., 2012; Jia et al., 2013). N6-methyladenosine (m^6^A) modification, which involves methylation of the N6 position of adenosine in RNA, is a common epigenetic transcriptional modification in eukaryotes. m^6^A is involved in multiple aspects of RNA biology and metabolism, including variable splicing, nuclear export, stability, and translation, with a particular impact on mRNA function (Motorin and Helm, 2022; Boulias and Greer, 2023). Additionally, m^6^A modifications have been identified in non-coding RNAs (lncRNAs, circRNAs, miRNAs, etc.) (Meyer et al., 2012; Lv et al., 2020). m^6^A modification is a dynamic and reversible epigenetic process (Fu et al., 2014; Meyer and Jaffrey, 2014; Roundtree et al., 2017a) mediated by three functional proteins, namely, methyltransferases (referred to as “writers”), demethylases (referred to as “erasers”), and m^6^A-binding proteins (referred to as “readers”), which are responsible for methylation, demethylation, and RNA binding, respectively (Wu et al., 2017; Flamand et al., 2023; Wang et al., 2023). Accumulating evidence suggests that m^6^A modification plays a critical role in regulating cellular mRNA function and is widespread among the genomic RNAs of DNA and RNA viruses, affecting viral infection (Zhao et al., 2020; Yu et al., 2021). The initial identification of m^6^A residues in all mRNAs was performed in the IAV, with eight m^6^A modification sites in the HA mRNA (Krug et al., 1976). Moreover, the distribution of m^6^A modification sites varies among different influenza virus mRNAs (Narayan et al., 1987). Recent studies have highlighted the significant role of m^6^A modification in regulating the gene expression and replication of IAV, although the precise regulatory mechanism involved remains unclear (Imam et al., 2020). This review focuses on recent studies that elucidate the molecular mechanisms of m^6^A modification and its underlying regulatory role in viral genomes and cellular antiviral responses during IAV infection. These findings provide a deeper understanding of the detailed interaction patterns between host proteins and viral proteins and offer new insights for future investigations into therapeutic interventions for IAV infection.

## 2 Molecular mechanisms of m^6^A modification

**Figure 1:**
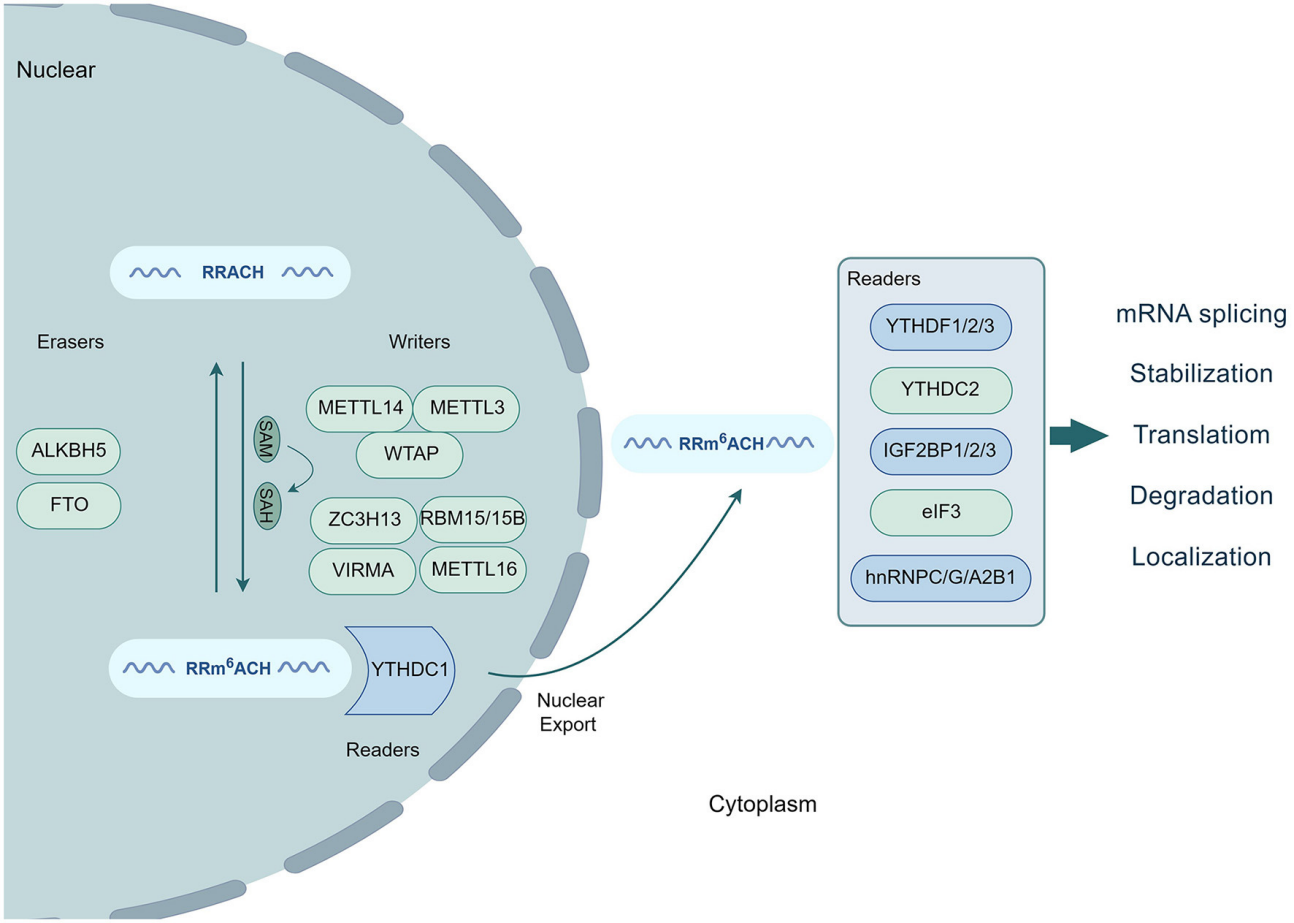
Process of m^6^A modification. The conserved RRACH motif of RNA undergoes methylation by writers (METTL3, METTL14, WTAP, ZC3H13, VIRMA, RBM15/15B, and METTL16) along with SAM conversion to SAH. This modification is reversibly controlled by erasers (FTO and ALKBH5) that demethylate RNA. Readers, such as YTHDC1/2, YTHDF1/2/3, IGF2BP1/2/3, eIF3, and hnRNPC/G/A2B1, recognize and bind to m^6^A-modified transcripts, influencing mRNA splicing, stabilization, translation, degradation, and localization.

Over the past decade, an increasing body of evidence has confirmed the involvement of m^6^A modification in regulating various physiological and pathological processes in mammalian cells. These processes include stem cell differentiation, cell reprogramming, stress response, circadian rhythm, DNA damage repair, metabolism, tumorigenesis, and inflammatory response (Shi et al., 2019; Wang et al., 2020; Huang W. et al., 2021). m^6^A modifications have been detected in different tissues and organs, with the highest levels observed in the brain, heart, and kidneys (Meyer et al., 2012). Three key proteins play vital roles in the m^6^A modification process. Methyltransferases mediate RNA methylation through stable multicomponent complexes in the nucleus (Murakami and Jaffrey, 2022; Sendinc and Shi, 2023). Specifically, methyltransferase-like 3 (METTL3) acts as the only subunit with catalytic activity, whereas its homolog, METTL14, serves as an allosteric activator that supports the catalytic activity of METTL3. These two proteins interact closely in the crystal structure, forming the stable heterodimer METTL3-METTL14 methyltransferase core complex, which plays a crucial role in substrate RNA recognition (Liu et al., 2014; Wang X. et al., 2016). In addition to the METTL3–METTL14 complex, the two CCCH-type zinc finger domains in the METTL3 structure also contribute to the complete enzymatic activity of the methyltransferase during the process of substrate RNA recognition and catalysis (Wang P. et al., 2016). In contrast, another homolog of METTL3, called METTL4, does not contain a catalytic domain and lacks catalytic activity. However, it can increase the catalytic efficiency of METTL3 (Luo et al., 2022). The m^6^A modification process is also regulated by the splicing factor Wilms tumor 1-associated protein (WTAP) (Ping et al., 2014). The m^6^A methyltransferase complex (MTC) consists of METTL3, METTL14, and WTAP. METTL3 contributes to the catalytic activity of MTC, whereas METTL14 recognizes and binds to its substrate RNA. WTAP is responsible for recruiting METTL3 and METTL14 to nuclear speckles for localization within subcellular structures (Sun et al., 2020). Additionally, several other proteins, including METTL16, Vir-like m^6^A methyltransferase-associated protein (VIRMA), zinc finger CCCH-type containing 13 (ZC3H13), and RNA-binding motif 15 (RBM15)/15B, are also involved in the RNA methylation process as part of the MTC (Zhang et al., 2019; Wang et al., 2021; Zhu W. et al., 2021; Lin et al., 2022; Satterwhite and Mansfield, 2022). Dominissini proposed a novel technique known as methylated RNA immunoprecipitation sequencing (MeRIP-seq). This innovative method combines immunoprecipitation using an anti-m^6^A affinity antibody with high-throughput RNA sequencing of the precipitated RNA fragments. The application of MeRIP-seq enabled Dominissini et al. to demonstrate, for the first time, that m^6^A modification sites exhibit greater sequence specificity than other regions (Dominissini et al., 2012). These m^6^A modification sites are not randomly distributed within transcripts; they are often found in the conserved RRACH motif of RNA (R = G/A, H = U/A/C) and are primarily enriched in stop codons and 3′-untranslated regions (3′-UTRs). Upon recognition and binding of the m^6^A modification sites on the targeted RNA, the MTC transfers methyl groups from S-adenosylmethionine (SAM) to the RNA, thereby influencing the functionality of both cellular- and virus-encoded mRNAs. Consequently, SAM is converted to S-adenosylhomocysteine (SAH) via demethylation. Conversely, demethylases are capable of removing methyl groups from the targeted RNA, effectively reversing the m^6^A modification process and dynamically regulating RNA methylation. Fat mass and obesity-associated protein (FTO) was the first demethylase identified to mediate RNA demethylation. Nuclear RNA serves as the primary substrate for FTO (Jia et al., 2011; Bayoumi and Munir, 2020). Furthermore, alkylation repair homolog protein 5 (ALKBH5) has been identified as another demethylase in mammals that exhibits demethylation activity comparable to that of FTO (Zheng et al., 2013). Both FTO and ALKBH5 regulate nuclear export, metabolism, and gene expression by removing methylation residues from mRNAs. During the process of RNA methylation, m^6^A-modified transcripts are specifically recognized and bound by various reader proteins, including the YTH domain family (YTHDC1–2 and YTHDF1–3) (Gokhale et al., 2016), IGF2BP1–3 (Müller et al., 2019), eIF3 (Choe et al., 2018), and hnRNPC/G/A2B1 proteins (Huang X.-T. et al., 2021). At the nuclear stage, m^6^A residues can bind to specific nuclear reader proteins, which can affect mRNA splicing, nuclear export, and other nuclear processes. Upon export to the cytoplasm, m^6^A residues bind to specific cytoplasmic reader proteins, thereby influencing the structural stability, degradation, translation, and localization of mRNA (Figure 1) (Zaccara et al., 2019). Using the linear amplification of complementary DNA ends and sequencing (LACE-seq) method (Su et al., 2021), Qiao demonstrated that the nucleus-localized RNA-binding protein (RBP) YTHDC1 specifically recognizes the m^6^A site through its C-terminal YTH domain. This recognition enables YTHDC1 to regulate the selective splicing and nuclear export of mRNA in skeletal muscle stem cells (Qiao et al., 2023). YTHDC1, in conjunction with hnRNPG, synergistically promotes mRNA splicing, regulates isomer diversity, and enhances mRNA output by binding to the THO nuclear export complex. These proteins also interact with the nuclear export adapter protein SRSF3, facilitating RNA binding to SRSF3 and nuclear RNA export factor 1 (NXF1), thereby mediating methylated mRNA export from the nucleus to the cytoplasm in HeLa cells. Deletion of YTHDC1 results in prolonged residence time for nuclear mRNA containing m^6^A, leading to the accumulation of transcripts in the nucleus and depletion in the cytoplasm (Roundtree et al., 2017b; Qiao et al., 2023).

In addition to YTHDC1, YTHDF1 also recruits argonaute family protein 2 (AGO2) via its YTH domain to promote the solvation and degradation of targeted mRNAs. However, the absence of YTHDF1 results in the formation of AGO2/RNA patches, delayed mRNA degradation, and cytoplasmic deposition (Li et al., 2022). The protein translation initiation factor EIF3C serves as a direct target for YTHDF1. m^6^A-modified EIF3C mRNA binds to YTHDF1, which then regulates EIF3C mRNA translation in an m^6^A-dependent manner. This promotes the synthesis of the oncogenic protein EIF3C, thereby facilitating tumor occurrence and metastasis in ovarian cancer (Liu et al., 2020). SUMOylation of YTHDF2 significantly enhances its mRNA binding ability. YTHDF2 selectively binds to m^6^A-methylated mRNAs to regulate mRNA degradation, such as increasing suppressor of cytokine signaling 2 (SOCS2) mRNA degradation, to promote METTL3-induced carcinogenesis (Chen et al., 2018; Hou et al., 2021). With respect to protein translation regulation, IGF2BP3 interacts with chromosome condensation 2 (RCC2) mRNA in an m^6^A-dependent manner to regulate its stability. This interaction contributes to the protein expression of RCC2 (Zhang N. et al., 2022) (Figure 1).

## 3 The role of m^6^A modification in IAV RNA transcripts

**Figure 2:**
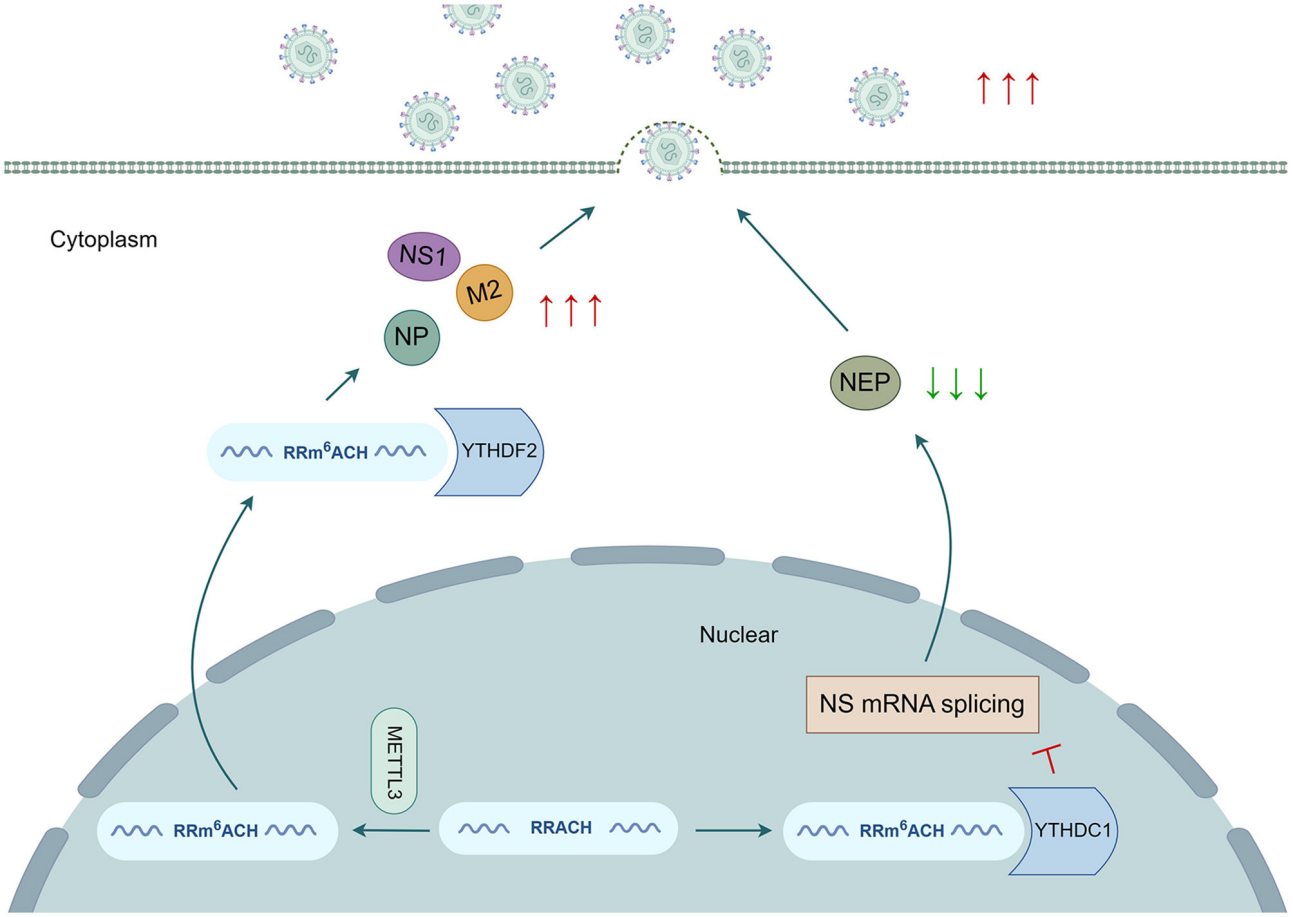
m^6^A modifications have an impact on the replication cycles of the influenza A virus. m^6^A modifications are involved in regulating various stages of IAV gene expression and replication. METTL3 and YTHDF2 enhance the expression of the NP, NS1, and M2 genes and the production of viral particles. Moreover, YTHDC1 reduces NEP gene expression by hindering NS mRNA splicing, thereby facilitating IAV replication.

IAV mRNA transcripts containing multiple internal m^6^A modification sites were initially detected, establishing a foundation for further investigation of viral epitranscriptomic regulation (Krug et al., 1976). The impact of m^6^A modification on virus gene expression and viral replication has been reported, as it regulates the splicing, stability, translation, and secondary structure of viral RNA (Figure 2). Previous research has indicated that the addition of m^6^A residues to IAV mRNA transcripts is crucial for promoting viral gene expression and replication. The absence of methyltransferase METTL3 significantly reduces the transcription of the NP, NS1, and M2 genes, as well as protein expression in IAV, leading to a decrease in the production of viral particles in a human lung epithelial cell line (A549 cells). Conversely, overexpression of the reading protein YTHDF2 has a noticeable positive effect on IAV gene expression and replication. However, the overexpression of YTHDF1 and YTHDF3 does not significantly affect IAV replication or the production of viral particles (Courtney et al., 2017). The role of YTHDF2 during IAV infection contradicts the promotion of intracellular mRNA degradation. The facilitation of intracellular mRNA degradation by YTHDF2 may diminish the transcription of host antiviral genes. As a result, the overexpression of YTHDF2 increases the expression level of viral mRNA and protein and the release of infectious virus particles, thereby promoting viral replication. By utilizing photoactivatable enhanced ribonucleoside crosslinking and immunoprecipitation (PAR-CLIP) and photo-crosslinking-assisted m^6^A sequencing (PA-m^6^A seq) technology (Hafner et al., 2010; Chen et al., 2015), the binding sites of the YTHDF protein were found to congregate into a sense mRNA open reading frame (ORF) and minus strand vRNA fragments encoding the viral structural proteins HA, NP, NA, and M, whereas their presence was limited to mRNA and vRNA fragments encoding the three subunits of RNA-dependent polymerase (PB1, PB2, and PA). These findings indicate the high selectivity of m^6^A modification in IAV RNA (Courtney et al., 2017; Bayoumi and Munir, 2021). Coupled with the enrichment of multiple highly conserved RRACH motifs in HA mRNA, the expression of HA mRNA and protein significantly decreases in m^6^A-deficient IAV mutants that target HA-modified sites without substantial effects on other viral mRNAs and proteins. Additionally, the pathogenicity and viral load of IAV mutants decrease. The m^6^A reader protein YTHDC1 has been reported to regulate IAV mRNA splicing by interacting with the NS1 protein in the nucleus. Increased expression of YTHDC1 was observed in A549 cells during the early and late stages of IAV infection. YTHDC1 directly targets and binds to the 3′ splicing site of NS mRNA, inhibiting the splicing of the NS segment dependent on the NS1 protein. Consequently, the expression of nuclear export protein (NEP) decreases, ultimately promoting the replication and pathogenicity of IAV, both *in vitro* and *in vivo* (Zhu Y. et al., 2023). In addition to the m^6^A modification, other modifications, such as 5-methylcytidine (m^5^C), N4-acetylcytidine (ac^4^C), and 2′ *O*-methylated nucleoside (N_m_), can influence various steps in the viral replication cycle. These modifications promote viral replication by increasing the stability and translation of viral mRNA and preventing host RNA-specific innate immune factor melanoma differentiation-associated protein 5 (MDA5) from recognizing and binding to viral RNA (Netzband and Pager, 2020; Tsai and Cullen, 2020) (Figure 2).

## 4 The regulation of m^6^A modification in IAV-infected cells

Innate immune signaling functions as the primary defense mechanism of host cells against viral infections. PRRs, such as RIG-I-like receptors (RLRs) and Toll-like receptors (TLRs), play crucial roles in identifying viral PAMPs. Upon recognition, these receptors trigger signaling cascade responses, leading to the production of interferon, inflammatory cytokines, and chemokines. These initial antiviral responses serve to combat viral infection (Iwasaki and Pillai, 2014; Onomoto et al., 2021; Zhou et al., 2021). Recent studies have highlighted the significant regulatory role of m^6^A modification in cellular mRNA function in response to IAV infection (Liu et al., 2019; Zhao et al., 2020). It has been confirmed that TANK-binding kinase 1 (TBK1) in the type I interferon signaling pathway can enhance the catalytic activity of METTL3 and promote the generation of interferon regulatory factor 3 (IRF3), thereby improving the antiviral immunity of host cells. In *Mettl3*-deficient mice infected with IAV, the production of IFN-β significantly decreased, inflammatory cells extensively infiltrated the lung tissue, and viral replication increased. These results indicate that METTL3 plays a crucial role in the host defense against viruses (Chen et al., 2022). Interestingly, another study revealed that m^6^A serves as a negative regulator of type I interferons. The methylation of IFN-β mRNA was mediated by METTL3 and YTHDF2 in THP-1 cells infected with various viruses, such as IAV, leading to decreased stability and increased degradation of IFN-β mRNA and resulting in reduced IFN-α and IFN-β production and downregulation of the host innate immune response. This process benefits viral replication and is conserved across various viral infections. However, following the depletion of METTL3 or YTHDF2 in IAV-infected cells, the expression of IFN-β and interferon-stimulated gene 15 (ISG15) mRNA significantly increased, while the expression of the IAV gene was inhibited (Winkler et al., 2019). METTL14 depletion increased both IFN-β mRNA production and stability in response to viral infection, leading to decreased virus protein expression and reproduction. In contrast, ALKBH5 depletion decreased the production of IFN-β mRNA without causing detectable changes in IFN-β mRNA decay, stimulating viral replication (Rubio et al., 2018). Current research shows that m^6^A can serve as either an inhibitor or a promoter for the expression of IFN-β mRNA. This conflict may be explained by the fact that the expression of IFN-β mRNA is significantly induced in the early stage of viral infection to activate the innate immune response, and starts to be degraded in the late stage to prevent inflammatory damage. Upon viral infection, METTL3 enhances the nuclear export and translation of antiviral signaling molecule mRNAs, such as IRF3, and promotes the induction of IFN-β and inflammatory cytokines against viral infection. Subsequently, m^6^A modifications (METTL3 and YTHDF2) promote the instability of IFN-β and ISG15 mRNA and control inflammation to maintain homeostasis. m6A modification has been confirmed to participate in the degradation of IFN-β mRNA in the termination of the innate response (Zhu J. et al., 2023). Furthermore, host cells actively reprogram cellular metabolism to resist viral infection in an interferon-independent manner through m^6^A modification. During viral infection, the enzymatic activity of ALKBH5 is reduced in host cells, which leads to the downregulation of the α-ketoglutarate dehydrogenase (OGDH)-itaconate pathway associated with viral infection mediated by YTHDF2. This subsequently inhibits viral replication (Liu et al., 2019). Overall, m^6^A modification plays an emerging role in the cellular defense against invading pathogens via virus–host interactions.

## 5 Anti-IAV strategies based on m^6^A

To date, numerous studies have demonstrated the effectiveness of small-molecule inhibitors targeting methylases and demethylases for antiviral therapy. One such inhibitor is 3-denitroadenosine (3-DAA), which inhibits SAH hydrolase. This inhibitor significantly reduces the m^6^A modification of viral RNA without affecting mRNA capping by depleting intracellular SAM and thus inhibiting the replication of various DNA and RNA viruses (Fustin et al., 2013; Kennedy et al., 2016). Consequently, 3-DAA shows promise as a broad-spectrum antiviral drug (Bray et al., 2000; Yanagi et al., 2022). However, it is important to note that 3-DAA also inhibits the methylation of histones and DNA, highlighting the need for more specific methylase inhibitors to eliminate off-target effects.

Another highly selective demethylase inhibitor is meclofenamic acid (MA), which primarily targets FTO rather than ALKBH5. MA competitively binds to FTO with m^6^A-methylated RNA in a dose-dependent manner, thus inhibiting the demethylation function of FTO (Huang et al., 2015). Furthermore, the metal protein nanoparticle GSTP1-MT3 (Fe^2+^) has demonstrated effective antiviral effects by increasing the expression of methyltransferases such as METTL14 and WTAP in virus-infected macrophages. GSTP1-MT3 (Fe^2+^) regulates m^6^A modification levels and inhibits the replication of the H1N1 (WSN) virus in various cells, providing potential avenues for the development of antiviral drugs that specifically target m^6^A modifications (Zhu X.-J. et al., 2021).

Previous studies have identified METTL3 as an efficient therapeutic target for the treatment of various tumors (Xu and Ge, 2022). For example, STM2457, the first bioavailable small-molecule inhibitor of METTL3, has been shown to significantly impede the development and progression of acute myeloid leukemia (AML) by directly binding to the enzymatic activity of METTL3 and inhibiting the catalytic activity of the METTL3/METTL14 MTC (Yankova et al., 2021). Importantly, compared to other RNA, DNA, and protein methyltransferases, STM2457 exhibits high selectivity for METTL3, thus reducing its off-target effects. However, it remains unknown whether small-molecule inhibitors targeting METTL3, such as STM2457, contribute to multiple viral infections and host immune responses.

In the future, the development of more selective and potent small inhibitors or drugs for m^6^A-related proteins may offer more effective therapeutic strategies to treat IAV infections and combat IAV evasion. Consequently, targeting m^6^A modifications may be a promising direction for future investigations of anti-IAV strategies.

## 6 Novel technologies used for RNA N6-methyladenosine methylation

### 6.1 MeRIP-seq

Dominissini proposed a novel approach called MeRIP-seq, which combines immunoprecipitation using an anti-m^6^A affinity antibody and high-throughput RNA sequencing of precipitated RNA fragments. Their research showed that m^6^A modification sites exhibit greater sequence specificity than other regions.

### 6.2 LACE-seq

The latest sequencing technology, LACE-seq, utilizes reverse transcription termination of immunopurified protein RNA complexes mediated by RBPs to amplify cDNA ends. This step is followed by high-throughput sequencing and analysis of the products. LACE-seq allows sensitive identification of binding sites and regulatory mechanisms between functional proteins and RNA.

### 6.3 PAR-CLIP

PAR-CLIP is a photoactivatable ribonucleoside-dependent technology used to identify m^6^A modification sites. This process involves the insertion of a photoactivatable ribonucleoside into RNA transcripts, leading to thymidine-to-cytidine transitions at the UV crosslinking sites of the anti-m^6^A affinity antibody and m^6^A-modified RNA. High-throughput sequencing of these transitions enables single-base resolution recognition of m^6^A modification sites.

### 6.4 PA-m^6^A-seq

PA-m^6^A-seq follows a procedure similar to that of PAR-CLIP but has the added ability to recognize single methylation sequences in a 23-base region and accurately identify transcriptome-wide m^6^A methylation sites.

## 7 Discussion

During m^6^A modification, the core MTC METTL3-METTL14 is responsible for initially recognizing the RNA substrate and transferring methyl groups to the RNA for methylation with the help of SAM. m^6^A-modified transcripts are then recognized and bound by reading proteins such as YTHDF1 and YTHDC1, which subsequently affect various aspects of the mRNA, including selective splicing processing, nuclear output, stability, translation, and localization. Concurrently, demethylases, such as FTO and ALKBH5, can remove methyl residues from RNA, reversing m^6^A methylation and dynamically regulating the balance of mRNA methylation. In the context of IAV infection, viral RNA undergoes specific modifications by methyltransferases and demethylases within infected host cells. m^6^A modification is involved in regulating multiple steps of the viral life cycle, particularly viral RNA transcription, and assembly, thereby playing a significant role in both viral infection and cellular antiviral responses. Notably, the replication of viral RNA has been shown to depend on m^6^A modification during IAV infection. The presence of METTL3 promotes the m^6^A modification of viral RNA, increasing the levels of viral mRNA and protein in addition to the release of virions, thereby enhancing viral replication and pathogenicity. Furthermore, m^6^A modification also regulates the mRNA transcription of various antiviral molecules associated with innate immune signaling pathways in host cells, thereby influencing splicing, stability, and translation in response to viral infection. By predominantly targeting the type I interferon signaling pathway, m^6^A modification has been identified as a regulator of the host immune response. The potential mechanism underlying this regulation is that m^6^A modification can promote mRNA nuclear export, stability, and translation of antiviral immune molecules (cGAS, IFI16, and STING), activate the downstream factor IRF3, induce the expression of IFN-β, and thus enhance the host antiviral immune response during the early stage of viral infection. However, at later stages, the m^6^A modification of IFN-β mRNA acts as a negative regulatory factor, promoting the degradation of IFN-β mRNA and alleviating IFN-β expression and inflammatory damage. As a result, the host's antiviral innate immune response is weakened. Additionally, the m^6^A modification of IAV RNA plays a crucial role in inhibiting the recognition and binding of the innate immune signal RIG-I to viral RNA, thus participating in viral immune evasion.

Current research indicates that m^6^A modification plays a crucial role in regulating the replication of IAV and the innate immune response to antiviral activity. However, there are numerous unresolved questions and controversies surrounding the precise regulation and facilitation of m^6^A modification proteins in cells infected by IAV. Therefore, it is crucial to further investigate the role of m^6^A modification in anti-IAV innate immunity and to explore the implications of various mapping techniques. Multiple studies have demonstrated that inhibitors targeting methyltransferases and demethylases can effectively disrupt IAV RNA replication by targeting m^6^A modifications. This discovery may provide innovative insights for the development of therapeutic interventions for IAV based on m^6^A modification.

## 8 Conclusion

m^6^A modification is the predominant form of epigenetic transcription modification in eukaryotes, and substantial evidence supports its significant role in regulating IAV infection. This review explores the mechanisms of three key proteins, methyltransferases, demethylases, and reading proteins, and the regulatory impact of m^6^A modification on IAV infection. These findings have the potential to identify valuable targets for anti-IAV strategies and serve as a foundation for implementing preventive or therapeutic measures.

## Author contributions

XL: Conceptualization, Investigation, Writing – original draft. WC: Funding acquisition, Supervision, Writing – review & editing. KL: Supervision, Writing – review & editing. JS: Supervision, Writing – review & editing, Conceptualization, Funding acquisition.
